# Occurrence and outcomes of retrobulbar haematoma in 2149 orbital fracture patients

**DOI:** 10.1007/s10006-025-01427-2

**Published:** 2025-07-10

**Authors:** Matilda Narjus-Sterba, Tero Puolakkainen, Linda Kokko, Hanna Thorén, Johanna Snäll

**Affiliations:** 1 Department of Oral and Maxillofacial Diseases, University of Helsinki and Helsinki University Hospital, Helsinki, Finland; 2 Department of Oral and Maxillofacial Surgery, Institute of Dentistry, University of Turku, Turku, Finland

**Keywords:** Orbital fractures, Retrobulbar haematoma, Orbital compartment syndrome, Blindness

## Abstract

**Purpose:**

We identified the occurrence of acute retrobulbar haematoma (RBH) across a complete range of orbital fractures and determined clinical and radiological findings that could predict permanent vision loss associated with RBH.

**Methods:**

A retrospective analysis was conducted on data from facial trauma patients, encompassing a comprehensive range of orbital fractures. The primary outcome variable was the presence of acute RBH that required surgical or medical treatment. The main predictor variables were the types of orbital fractures. We collected data on symptoms, clinical and radiological findings, treatment, and instances of vision loss to assess the relationship between these factors and vision impairment.

**Results:**

Of the 2149 patients with orbital fractures, 28 (1.3%) presented with acute RBH, including two bilateral haematomas, bringing the total number of RBHs to 30. Specific injury mechanisms were statistically associated with RBH (*p* = 0.005), with high-energy injuries being the most frequent cause. The prevalence of RBH was higher in bilateral than unilateral fractures (3.1% vs. 1.1%) (*p* = 0.023). Among unilateral fractures, RBH was most strongly linked to orbital roof and rim fractures. Tenting or tuliped-like appearance on computed tomography and absence of pupillary light reflex were more common in patients with permanent vision loss. Type of surgical approach did not affect visual outcome.

**Conclusion:**

Acute RBH appears to occur more frequently in patients with orbital fractures than previously reported. Clinicians managing facial fractures need to be trained to diagnose RBH promptly and identify indicators of potential vision loss, particularly tenting on radiological images and changes in pupillary reflex.

## Introduction

Retrobulbar hematoma (RBH) is a rare but serious condition, with an estimated incidence of 0.45–0.6% in orbital fractures [1, 2]. However, only a fraction of RBH progresses to orbital compartment syndrome (OCS), requiring intervention. Current evidence suggests that RBH is caused by arterial bleeding into the confined retrobulbar space, typically associated with surgical complications or, more commonly, with non-displaced fractures of the orbital walls, particularly the orbital roof [1, 3].

Common symptoms of RBH include pain, proptosis, conjunctival oedema, restricted extraocular movement, increased intraorbital pressure (IOP), ocular firmness, and, most critically, vision loss. Vision impairment has been reported in up to 22% of RBH cases [4], although the underlying mechanisms remain unclear. Blood accumulation in the orbit leads to an increase in IOP, but the link between this rise and permanent vision loss is not fully understood. Some studies suggest that elevated IOP disrupts perfusion, resulting in ischaemia of the optic nerve and retina, leading to vision loss [5]. However, other reports document visual impairment occurring despite the absence of elevated IOP [6]. It is important to note that visual impairment may not manifest immediately following a traumatic event [7–9]. Timely intervention is critical, as patients may experience irreversible vision loss within 60 to 120 min of RBH formation [2, 10, 11].

Despite the potential for severe outcomes associated with RBH, standardized treatment protocols have not yet been established. Current management primarily focuses on orbital decompression to relieve pressure and preserve ocular and optic nerve perfusion. While some studies suggest that medical therapy alone can prevent vision loss, surgical interventions—such as lateral canthotomy or cantholysis, with or without haematoma evacuation—remain the preferred approach for vision-threatening RBH [1, 12–14]. Medical therapy is often used as an adjunct to surgery.

The emergent nature and rarity of RBH have limited the number of studies addressing associated fracture types and management strategies. This study retrospectively examines the incidence of RBH among facial trauma patients across all orbital fracture types, exploring variations in its occurrence based on fracture patterns and patient demographics. Additionally, we assess the frequency of vision loss and analyse potential explanatory variables influencing its development. Our hypothesis is that the incidence of acute RBH exceeds previously reported rates and that specific clinical and radiological findings could ultimately serve as predictors of impending permanent vision impairment.

## Materials and methods

### Study design

Retrospective data were gathered from the electronic medical database of all facial fracture patients treated at the tertiary trauma unit, Töölö Hospital Emergency Department, Helsinki University Hospital (HUH), Helsinki, Finland, over a nine-year period, from 1 January 1, 2013 to 31 October 2020. Patients’ medical records were reviewed, and the study data were collected from the records.

The study included patients with isolated orbital fractures and those with fractures extending into the orbit, as confirmed by computed tomography (CT) imaging. We retrospectively assessed the incidence of RBH in these cases and investigated potential factors contributing to its onset.

Data collected included patient age, sex, mechanism of injury, type of orbital fracture, and presence of clinically significant RBH. We also gathered information on RBH-specific symptoms, clinical and radiological findings, time intervals from injury to CT diagnosis and from diagnosis to treatment, treatment details, and resulting vision outcome. Furthermore, we analysed whether these variables contribute to or could predict RBH-related visual impairment.

### Study variables

#### RBH occurrence

The outcome variable for this study was the presence of acute RBH, defined as a RBH requiring surgical intervention or medical treatment with high-dose glucocorticoids and ocular medications used to manage intraocular pressure.

The primary predictor variable was the type of orbital fracture, categorized as unilateral or bilateral. Unilateral fractures were classified into subtypes according to the specific orbital structures involved, which served as additional predictor variables. The subtypes were categorized as follows: unilateral blow-out fractures (involving the orbital floor, medial wall, or both), unilateral zygomatico-orbital fractures, unilateral orbital roof and rim fractures, and unilateral combined orbital fractures (comprising any other combinations not listed above).

The explanatory variables included age, sex, and mechanism of injury. A comprehensive overview of these variables is shown in Table 1.

#### Patients’ symptoms, clinical and radiological findings, and details of treatment

The symptoms, along with the clinical and radiological findings of RBH patients, were systematically reviewed. We examined how these factors, delays in diagnosis and treatment, and specific treatment methods relate to vision loss. Tables 2 and 3, and 4 provide detailed descriptions of these variables.

Radiological findings were obtained from the initial CT images taken after the injury. When necessary, the primary radiologist’s report was supplemented by a consultation with a maxillofacial surgeon specialized in facial trauma (J.S.). CT images of patients with retrobulbar haematoma were re-evaluated by authors J.S. and M.N-S. The maximum haematoma thickness was measured in millimetres (mm). In cases of unilateral fractures, radiological proptosis was assessed in the axial view at the orbital rim level, along the cross-sectional line of the globe, as previously described by Zimmerer et al. [15]. The distance from the outer part of the globe to the cross-sectional line was measured and compared with the unaffected side, and the difference was recorded in millimetres.

#### Persistent vision loss

Patients with surgically treated RBH whose visual acuity was possible to assess postoperatively were further analysed. The primary outcome variable was persistent vision loss, classified as moderate or worse visual impairment in the affected eye. The severity of visual impairment was classified according to the International Statistical Classification of Diseases [16].

#### Statistical analyses

Statistical analyses were conducted using SPSS Statistics version 28.0 (IBM, N.Y., USA). The Kruskal-Wallis test was used to assess the differences in age distribution between patients with and without RBH. To evaluate associations between RBH and other categorical explanatory and predictor variables, Pearson’s Chi-squared test and Fisher’s exact test were employed. A *p*-value of less than 0.05 was considered statistically significant.

### Ethical approval

This study was conducted in accordance with the principles of the Declaration of Helsinki. Institutional review board approval was obtained for this retrospective study (HUS/356/2017).

## Results

### Patients and orbital fracture types

Throughout the study period, records from 2149 facial trauma patients with orbital fractures were manually analysed. Age ranged from 3.3 to 102.5 years, with a median of 49.2 years. Males comprised 70.2% of patients. Ground-level falls (30.8%) and assault (26.2%) were the leading causes of injury. Unilateral fractures accounted for 89.4% of cases, with zygomatico-orbital fractures (42.5%) and blow-out fractures (41.0%) being the most common.

### Occurrence of RBH

Out of 2149 patients with orbital fractures, 28 (1.3%) developed a total of 30 acute RBHs. Although not statistically significant, patients with RBH tended to be older than those without it (median age 58.1 vs. 49.0 years). The occurrence of RBH varied significantly by mechanism of injury (*p* = 0.005), with motor vehicle accidents and high-energy falls each contributing the highest rates at 2.7% (Table 1).

The prevalence of RBH was higher in patients with bilateral fractures than in patients with unilateral fractures (3.1% vs. 1.1%) (*p* = 0.023). Variation in RBH frequency was observed among the different types of unilateral fractures (*p* = 0.004); RBH occurred in 4.3% of patients with orbital roof/rim fractures, 1.3% with blow-out fractures, 0.6% with combined fractures, and 0.5% with zygomatico-orbital fractures.

### Symptoms and clinical and radiological findings in patients with RBH

Detailed findings from the 30 RBHs are summarized in Table 2. Surgical intervention was required in 24 cases, while 6 received only local medical treatment with topical intraocular pressure-lowering medications (brinzolamide or dorzolamide). Eleven patients were on anticoagulants, accounting for 36.7% of the 30 RBHs, as none had bilateral hematoma.

**Fig. 1:**
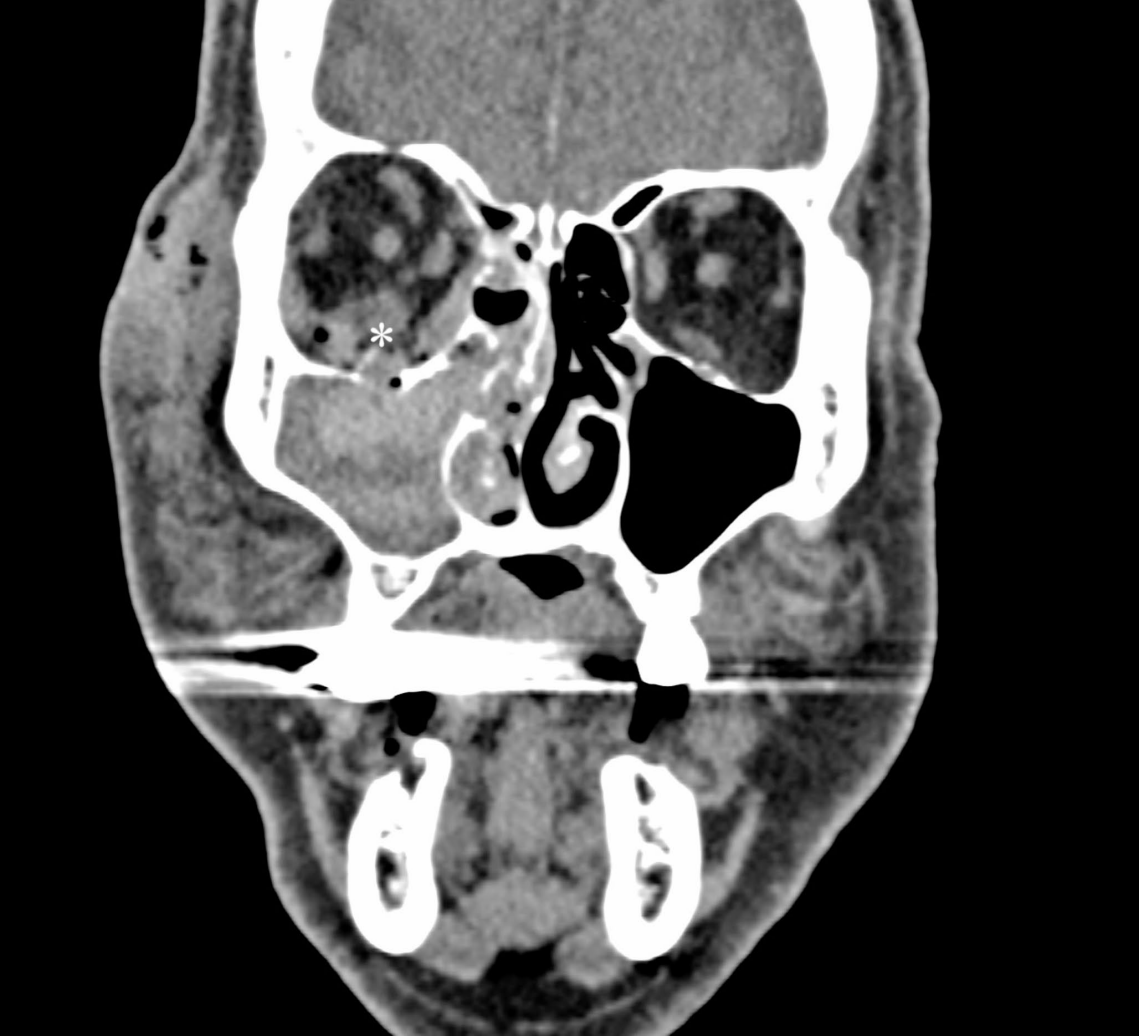
A 66-year-old man was assaulted and subsequently evaluated for facial injuries in the emergency department. Imaging revealed a right-sided blow-out fracture involving the floor and medial wall, accompanied by a 7-mm-thick haematoma in the fragmented orbit (*)

Radiological analyses revealed that RBHs were most frequently located in the roof (23.3%) and floor (16.7%) of the orbit, with lower frequencies observed in the medial and lateral walls (6.7% each). In 46.7% of cases, hematomas were present in multiple locations (Fig. 1).

Tenting was identified on 9 CT images (30.0%). The maximum hematoma thickness ranged from 3 to 20 mm (median 7.0 mm). Radiological proptosis measurements varied from 1 to 10 mm (median 5.0 mm).

Clinically, loss of pupillary light reflex was recorded in 22 cases (73.3%). Intraocular pressure measurements were available for 22 cases, ranging from 18 to 66 mmHg (median 36.2 mmHg). Restricted eye movement was noted in 14 cases (46.7%) and could not be assessed in 12 (40.0%).

### Vision loss in patients

Of the 30 RBH cases, 7 (23.3%) resulted in persistent vision loss; all these RBHs were treated surgically. Three patients experienced total blindness, three had severe visual impairment, and one had minor impairment. Three patients died before a final vision assessment could be completed, including one patient with bilateral haematomas. Ophthalmologic consultation was provided for 24 patients, covering a total of 26 RBHs, including all 7 patients who developed persistent vision loss.

### Predictors for persistent vision loss in RBH patients treated surgically

**Fig. 2:**
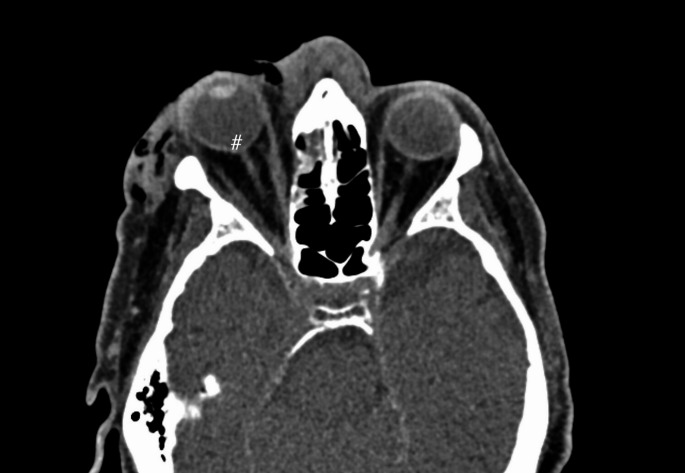
Clinical findings in the patient case shown in Fig. 1 included slight stiffness of the right pupil, restricted eye movements, increasing pain, and elevated intraocular pressure (right: 48.4 mmHg, left: 22.1 mmHg). These symptoms indicated a wide haematoma causing 8 mm proptosis and tenting of the optic nerve (#). An emergency canthotomy was performed in the maxillofacial emergency unit to reduce intraorbital pressure 5 h after the trauma, along with topical ocular medication. Vision remained normal during follow-up

For the 20 surgically treated RBH patients with assessable postoperative visual acuity, potential predictors of vision loss were analysed (Tables 3 and 4). Tenting on CT imaging (Fig. 2) was strongly associated with permanent vision impairment. Among the seven patients exhibiting the tenting sign, five (71.4%) experienced irreversible vision loss. Other variables demonstrated less distinct relationships, as they were also prevalent among RBH patients without vision impairment; for example, pupil stiffness was present in 58.8%, and restricted eye movement was observed in 55.6% of those patients. However, all patients with permanent visual impairment exhibited an absent pupillary reflex, and also restricted eye movement whenever it was assessable.

RBHs in patients with visual loss exhibited slightly greater proptosis (median 7.0 mm vs. 5.0 mm) and thickness (median 9.0 mm vs. 7.0 mm) than those without vision changes.

The time range from trauma to CT imaging (1.2 to 9.5 h) and from trauma to surgery (1.9 to 27.3 h) was prolonged. Treatment timing in patients with visual impairment was marginally more delayed than in those without (median 8.4 h vs. 6.5 h), but no strong correlation was identified. No association was found with the type of surgical approach, as cases were evenly distributed across methods. Vision loss was not linked to anticoagulant medication, as only two patients on anticoagulants experienced permanent visual loss. Similarly, no correlation was found with high-dose glucocorticoid administration; among the six patients treated with glucocorticoids, two sustained permanent visual impairment.

## Discussion

This retrospective study examined the incidence of RBH in a large dataset of facial trauma patients, focusing on variations across fracture types and patient demographics. Results revealed a markedly higher rate of acute RBH in orbital fracture patients (1.3%) than previously reported (0.45–0.6%) [1, 2]. Changes in pupillary reflex and tenting on CT imaging were identified as potential indicators for vision loss.

Fracture type, mechanism of injury, and patient age have been linked to a higher incidence of RBH. Especially fractures involving the orbital roof have shown a strong association [1, 3], possibly due to their fissural nature, which obstructs blood drainage and raises IOP. In this study, RBH was most frequent in bilateral orbital fractures —likely because of the higher prevalence of high-energy trauma— and unilateral orbital roof fractures, although cases were observed across all fracture types. Importantly, RBHs can occur across all age groups. In this study, the youngest patient to undergo immediate haematoma evacuation was 9 years old.

Only a small number of RBH progress to vision-threatening OCS. The exact mechanism behind vision loss remains unclear. Recovery after prompt orbital decompression suggests that both IOP and timely intervention play critical roles. Animal studies have shown that once a critical pressure threshold is exceeded, the duration of pressure may be more detrimental to neuronal survival than its intensity [17]. Intervention within 60 to 120 min of RBH onset is considered crucial [2, 10, 11]. In our study, patients who experienced vision impairment received slightly delayed treatment (median 8.4 h vs. 6.5 h). However, RBH may not develop immediately after injury [7–9], complicating the determination of the treatment window. In addition, vision loss has been documented in RBH patients even without elevated pressure [6]. Some studies also report poor vision recovery despite timely intervention within the recommended two-hour window [18]; conversely, others document recovery even with prolonged delays [19–21]. It is, therefore, difficult to establish general threshold values for treatment timing.

Identifying clinical and radiological markers of vision impairment is crucial for early recognition of at-risk patients. Christie et al. [4] reported that among OCS patients, 44% experienced vision changes, 40% had proptosis, 35% had pupillary abnormalities, 30% showed restricted eye movement, 16% had elevated IOP, and 15% reported pain. Popat et al. [22] suggested that RBH might be predicted by three or more of the following signs: pain, proptosis, chemosis, diplopia, subconjunctival haemorrhage, elevated IOP, tense globe, reduced vision, restricted movement, or loss of pupillary reflex. However, the clinical relevance of many of these indicators is debated. Signs like chemosis, subconjunctival haemorrhage, diplopia, vision changes, and restricted movement lack specificity, as they are common in orbital fractures but often unrelated to RBH. Pain is also unreliable, as many patients present with severe trauma or are unconscious on admission.

Similarly, IOP assessment has its limitations. Retrobulbar pressure does not always correlate with IOP [23], and many emergency departments lack the necessary equipment for accurate measurement. Readings obtained with improper techniques have been reported to vary even by 50–100% [24]. When IOP measurement is infeasible, globe palpation is sometimes used, although it remains highly subjective and imprecise.

In unconscious patients, the absence of a pupillary response is considered a strong indicator [1] and our results support this, as all patients with vision loss exhibited non-reactive, light-stiff pupils. However, systemic conditions, intracranial injuries, or the effects of substances and medications may complicate diagnosis. Proptosis is another indicator commonly used for unconscious patients, but it does not directly correlate with IOP. Other causes —such as bony displacement into the orbit (blow-in fracture) [25], orbital bleeding [26], or retrobulbar swelling [1, 27] — must also be considered.

The role of CT imaging in diagnostics remains debated. Some studies advocate for clinical evaluation alone [3, 28], while others highlight the value of CT in identifying haematoma characteristics [29] and associated findings such as orbital fractures and intracranial injuries. In our study, tenting—a tulip-like appearance on CT—emerged as another significant indicator of permanent visual impairment, observed in five of the seven patients with vision loss. Previous studies have similarly recognized the tulip-like bulb [15, 30, 31] as a key radiological finding in acute RBH.

Haematoma location is also thought to be a significant factor in determining vision loss; however, the rarity of RBHs presents challenges for studying RBH confined to specific areas. Reddy et al. [32] reported that intraconal haematomas and those adjacent to the optic nerve were associated with poor visual outcome. In this study, vision loss was most prevalent among patients with haematomas located in the superior orbit or at multiple sites.

The primary limitation of this study is the small number of RBH patients, which restricted the statistical analysis. Additionally, certain variables, such as pain, could not be assessed retrospectively. Despite these limitations, this study is one of the most comprehensive investigations of RBH in orbital fracture patients, with an extensive nine-year study period and a large dataset encompassing all facial fracture patients treated at a tertiary trauma centre during this time. To prevent future RBH-related visual impairments, it is crucial to enhance awareness of acute RBH and the critical predictors—particularly tenting on CT scans and changes in pupillary reflex—among clinicians. Further multicentre studies are needed to better understand the underlying causes of RBH-related vision loss, improve the identification of at-risk patients, and establish standardized treatment protocols for those requiring urgent intervention.

**Table 1:** Associations between explanatory and predictor variables and occurrence of retrobulbar haematoma in 2149 orbital fracture patients

		Patients with RBH	% of *n*	Patients without RBH	% of *n*	
		*n*		*n*		
	2149	28	1.3	2121	98.7	
						***p***-**value**
**Age (years)**						0.165^1^
Median (range)	49.2 (3.3, 102.5)	58.1 (9.1, 92.2)		49.0 (3.3, 102.5)		
Interquartile range	35.8	41.5		35.7		
**Sex**	**n (%)**	**n (%)**		**n (%)**		0.999^2^
Male	1508 (70.2)	20 (71.4)	1.3	1488 (70.2)	98.7	
Female	641 (29.8)	8 (28.6)	1.2	633 (29.8)	98.8	
**Mechanism of injury**						0.005^3^
Fall on ground level	661 (30.8)	6 (21.4)	0.9	655 (30.9)	99.1	
Assault	562 (26.2)	6 (21.4)	1.1	556 (26.2)	98.9	
Non-motorized vehicle	275 (12.8)	0 (0.0)	0.0	275 (13.0)	100.0	
Fall from height and fall from stairs	263 (12.2)	7 (25.0)	2.7	256 (12.1)	97.3	
Motor vehicle accident	186 (8.7)	5 (17.9)	2.7	181 (8.5)	97.3	
Struck by object	152 (7.1)	2 (7.1)	1.3	150 (7.1)	98.7	
Other/unknown	45 (2.1)	1 (3.6)	2.2	44 (2.1)	97.8	
Firearm	5 (0.2)	1 (3.6)	20.0	4 (0.2)	80.0	
**Orbital fracture type**						0.023^3^
Bilateral fracture	227 (10.6)	7 (25.0)	3.1	220 (10.4)	96.9	
Unilateral fracture	1922 (89.4)	21 (75.0)	1.1	1901 (89.6)	98.9	
**Unilateral fracture subtype**						0.004^3^
Unilateral zygomatico-orbital	816 (42.5)	4 (19.0)	0.5	812 (42.7)	99.5	
Unilateral blow-out	788 (41.0)	10 (47.6)	1.3	778 (40.9)	98.7	´
Unilateral combined	179 (9.3)	1 (4.8)	0.6	178 (9.4)	99.4	
Unilateral orbital roof/rim	139 (7.2)	6 (28.6)	4.3	133 (7.0)	95.7	

RBH = retrobulbar haematoma

^1^Kruskal-Wallis rank sum test

^2^Pearson’s Chi-squared test

^3^Fisher’s Exact test for count data

**Table 2:** Treatment approaches, symptoms, and clinical and radiological findings in 30 cases of surgically and medically treated retrobulbar haematoma

	*n*	% of *n*
**Antociagulant medication**		
Yes	11	36.7
No	19	63.3
**Treatment approach**		
Surgical	24	80.0
Exclusively medical	6	20.0
**Orbital site of haematoma (radiological view)**		
Roof	7	23.3
Combined	14	46.7
Floor	5	16.7
Medial	2	6.7
Lateral	2	6.7
**Tulip-like bulbus (radiological view)**		
Yes	9	30.0
No	21	70.0
**Maximum thickness of haematoma**,** mm (radiological view)**		
Range	3 to 20	
Mean	7.7	
Median	7.0	
**Proptosis**, mm (radiological view) *		
Range	1 to 10	
Mean	5.0	
Median	5.0	
**Eye pressure before treatment**, mmHg **		
Range	18 to 66	
Mean	35.8	
Median	36.2	
**Pupil light stiffness**		
Yes	22	73.3
No	7	23.3
Not available	1	3.3
**Restricted eye movements**		
Yes	14	46.7
No	4	13.3
Not available	12	40.0
**Persistent vision loss**		
Yes	7	23.3
No	19	63.3
Not available	4	13.3

* Radiological proptosis could not be evaluated in two patients due to bilateral haematoma

** Information on eye pressure available for 22 patients

**Table 3:** Association between presurgical variables and persistent vision loss in patients with surgically treated retrobulbar haematoma*

	All		Patients with persistent vision loss *n* = 7			Patients without persistent vision loss *n* = 13	
	*n*			% of *n*			% of *n*
**Antociagulant medication**							
Yes	8		2	25.0		6	75.0
No	12		5	41.7		7	58.3
**Orbital site of haematoma (radiological view)**							
Combined	7		3	42.9		4	57.1
Roof	6		3	50.0		3	50.0
Floor	4		1	25.0		3	75.0
Medial	2		0	0.0		2	100.0
Lateral	1		0	0.0		1	100.0
**Tulip-like bulbus (radiological view)**							
Yes	7		5	71.4		2	28.6
No	13		2	15.4		11	84.6
**Maximum thickness of haematoma**,** mm (radiological view)**							
Range	3 to 20		6 to 20			3 to 15	
Mean	8.7		11.3			7.3	
Median	7.0		9.0			7.0	
**Proptosis**, mm (radiological view)**							
Range	2 to 10		2 to 10			2 to 8	
Mean	5.5		6.8			4.8	
Median	6.0		7.0			5.0	
**Eye pressure before treatment**,** mmHg**							
Range	18 to 66		32.5 to 50.0			18 to 66	
Mean	38.9		40.0			37.8	
Median	38.5		40.0			35.0	
**Pupil light stiffness**							
Yes	17		7	41.2		10	58.8
No	2		0	0.0		2	100.0
Not evaluated	1		0	0.0		1	100.0
**Restricted eye movements**							
Yes	9		4	44.4		5	55.6
No	3		0	0.0		3	100.0
Not evaluated	8		3	37.5		5	62.5

* Data on final visual acuity available for 20 patients

** Radiological proptosis could not be evaluated in two patients due to bilateral haematoma

**Table 4:** Time span from injury to surgery and treatment details of patients* with surgically treated retrobulbar haematoma

	All		Patients with persistent vision loss				Patients without persistent vision loss		
**Time span, hours**									
**From trauma to treatment**									
Range	1.9 to 27.3			4.3 to 27.0				1.9 to 27.3	
Mean	9.0			10.9				9.9	
Median	6.9			8.4				6.5	
**From trauma to CT**									
Range	1.2 to 9.5			1.2 to 9.5				1.3 to 5.5	
Mean	3.1			3.9				2.7	
Median	2.4			2.4				2.4	
**From CT to treatment**									
Range	0.4 to 25.8			1.8 to 25.8				0.4 to 24.8	
Mean	7.7			7				7.2	
Median	3.6			4.6				3.2	
				**n**	**% of n**			**n**	**% of n**
**Surgical treatment**									
Canthotomy	7			2	28.6			5	71.4
Canthotomy + evacuation	6			3	50.0			3	50.0
Exclusively evacuation	3			1	33.3			2	66.7
Cantholysis + evacuation	1			1	100.0			0	0.0
Cantholysis	3			0	0.0			3	100.0
**High-dose glucocorticoid**									
Yes	5			2	40.0			3	60.0
No	15			5	33.3			10	66.7

* Data on final visual acuity available for 20 patients

CT = computed tomography

## Data Availability

The data analyzed during the current study are not publicly available due to confidentiality reasons; however, the corresponding author will provide them upon reasonable request.

## References

[1] Gerbino G, Ramieri GA, Nasi A (2005) Diagnosis and treatment of retrobulbar haematomas following blunt orbital trauma: A description of eight cases. Int J Oral Maxillofac Surg 34(2):127–131. 10.1016/j.ijom.2004.05.001 15695039 10.1016/j.ijom.2004.05.001

[2] Hislop WS, Dutton GN, Douglas PS (1996) Treatment of retrobulbar hemorrhage in accident and emergency departments. Br J Oral Maxillofac Surg 34(4):289–292. 10.1016/s0266-4356(96)90004-2 8866062 10.1016/s0266-4356(96)90004-2

[3] Kondoff M, Nassrallah G, Ross M, Deschênes J (2019) Incidence and outcomes of retrobulbar hematoma diagnosed by computed tomography in cases of orbital fracture. Can J Ophthalmol 54(5):606–610. 10.1016/j.jcjo.2019.01.006 31564352 10.1016/j.jcjo.2019.01.006

[4] Christie B, Block L, Ma Y, Wick A, Afifi A (2018) Retrobulbar hematoma: A systematic review of factors related to outcomes. J Plast Reconstr Aesthet Surg 71(2):155–161. 10.1016/j.bjps.2017.10.025 29239798 10.1016/j.bjps.2017.10.025

[5] Ord RA (1981) Post-operative retrobulbar haemorrhage and blindness complicating trauma surgery. Br J Oral Surg 19(3):202–207. 10.1016/0007-117x(81)90005-6 6945123 10.1016/0007-117x(81)90005-6

[6] Wood CM (1989) The medical management of retrobulbar haemorrhage complicating facial fractures: A case report. Br J Oral Maxillofac Surg 27(4):291–295. 10.1016/0266-4356(89)90040-5 2504275 10.1016/0266-4356(89)90040-5

[7] Ghufoor K, Sandhu G, Sutcliffe J (1998) Delayed onset of retrobulbar haemorrhage following severe head injury: A case report and review. Injury 29(2):139–141. 10.1016/s0020-1383(97)00129-0 10721409 10.1016/s0020-1383(97)00129-0

[8] Chen YA, Singhal D, Chen YR, Chen CT (2012) Management of acute traumatic retrobulbar haematomas: A 10-year retrospective review. J Plast Reconstr Aesthetic Surg 65(10):1325–1330. 10.1016/j.bjps.2012.04.037 10.1016/j.bjps.2012.04.037 22717974

[9] Hislop WS, Dutton GN (1994) Retrobulbar haemorrhage: can blindness be prevented? Injury 25. 10663–665. 10.1016/0020-1383(94)90009-4 10.1016/0020-1383(94)90009-4 7829190

[10] Hayreh SS, Kolder HE, Weingeist TA (1980) Central retinal artery occlusion and retinal tolerance time. Ophthalmology 87(1):75–78. 10.1016/s0161-6420(80)35283-4 6769079 10.1016/s0161-6420(80)35283-4

[11] Colletti G, Valassina D, Rabbiosi D, Pedrazzoli M, Felisati G, Rossetti L, Biglioli F, Autelitano L (2012) Traumatic and iatrogenic retrobulbar hemorrhage: an 8 patient series. J Oral Maxillofac Surg 70(8):464–468. 10.1016/j.joms.2012.05.007 10.1016/j.joms.2012.05.007 22793960

[12] Goodall KL, Brahma A, Bates A, Leatherbarrow B (1999) Lateral canthotomy and inferior cantholysis: an effective method of urgent orbital decompression for sight threatening acute retrobulbar haemorrhage. Injury 30(7):485–490. 10.1016/s0020-1383(99)00137-0 10707216 10.1016/s0020-1383(99)00137-0

[13] Winterton JV, Patel K, Mizen KD (2007) Review of management options for a retrobulbar hemorrhage. J Oral Maxillofac Surg 65(2):296–299. 10.1016/j.joms.2005.11.089 17236937 10.1016/j.joms.2005.11.089

[14] Yung CW, Moorthy RS, Lindley D, Ringle M, Nunery WR (1994) Efficacy of lateral canthotomy and cantholysis in orbital hemorrhage. Ophthal Plast Reconstr Surg 10(2):137–141. 10.1097/00002341-199406000-00012 8086363 10.1097/00002341-199406000-00012

[15] Zimmerer R, Schattmann K, Essig H, Jehn P, Metzger M, Kokemüller H, Gellrich N-C, Tavassol F (2014) Efficacy of transcutaneous transseptal orbital decompression in treating acute retrobulbar hemorrhage and a literature review. Craniomaxillofac Trauma Reconstr 7(1):17–26. 10.1055/s-0033-1356754 24624253 10.1055/s-0033-1356754 PMC3931771

[16] International Statistical Classification of Diseases and Related Health Problems (2010) chapter VII, H54

[17] Göçer AI, Ildan F, Haciyakupoglu S, Tuna M, Bağdatoğlu H, Polat S, Çetinalp E, Aksoy K (1996) The effect of immediate decompression on the optic nerve in retrobulbar hematoma. Neurosurg Rev 19(3):169–173. 10.1007/BF00512047 8875505 10.1007/BF00512047

[18] Dixon JL, Beams OK, Levine BJ, Papas MA, Passarello BA (2020) Visual outcomes after traumatic retrobulbar hemorrhage are not related to time or intraocular pressure. Am J Emerg Med 38(11):2308–2312. 10.1016/j.ajem.2019.10.024 31784392 10.1016/j.ajem.2019.10.024

[19] Pamukcu C, Odabasi M (2015) Acute retrobulbar haemorrhage: an ophthalmologic emergency for the emergency physician. Ulus Travma Acil Cerrahi Derg 21(4):309–314. 10.5505/tjtes.2015.16768 26374422 10.5505/tjtes.2015.16768

[20] Vassallo S, Hartstein M, Howard D, Stetz J (2002) Traumatic retrobulbar hemorrhage: emergent decompression by lateral canthotomy and cantholysis. J Emerg Med 22(3):251–256. 10.1016/s0736-4679(01)00477-2 11932087 10.1016/s0736-4679(01)00477-2

[21] Carrim ZI, Anderson IWR, Kyle PM (2007) Traumatic orbital compartment syndrome: importance of prompt recognition and management. Eur J Emerg Med 14(3):174–176. 10.1097/MEJ.0b013e3280b17e49 17473616 10.1097/MEJ.0b013e3280b17e49

[22] Popat H, Doyle PT, Davies SJ (2007) Blindness following retrobulbar haemorrhage—it can be prevented Br. J Oral Maxillofac Surg 45(2):163–164. 10.1016/j.bjoms.2005.06.028 10.1016/j.bjoms.2005.06.028 16099557

[23] Kratky V, Hurwitz JJ, Avram DR (1990) Orbital compartment syndrome. Direct measurement of orbital tissue pressure: 1. Technique. Can J Ophthalmol 25(6):293–297 2249165

[24] Erickson BP, Garcia GA (2020) Evidence-based algorithm for the management of acute traumatic retrobulbar haemorrhage. Br J Oral Maxillofac Surg 58(9):1091–1096. 10.1016/j.bjoms.2020.05.026 32546417 10.1016/j.bjoms.2020.05.026

[25] Gabrielli MA, Vieira EH, Gabrielli MF, Barbeiro RH (1997) Orbital roof blow-in fracture: report of a case. J Oral Maxillofac Surg 55(12):1475–1478. 10.1016/s0278-2391(97)90654-6 9393410 10.1016/s0278-2391(97)90654-6

[26] Burkat CN, Lemke BN (2005) Retrobulbar hemorrhage: inferolateral anterior orbitotomy for emergent management. Arch Ophthalmol 123(9):1260–1262. 10.1001/archopht.123.9.1260 16157809 10.1001/archopht.123.9.1260

[27] Perry M (2008) Acute proptosis in trauma: retrobulbar hemorrhage or orbital compartment syndrome—does it really matter? J Oral Maxillofac Surg 66(9):1913–1920. 10.1016/j.joms.2008.04.012 18718400 10.1016/j.joms.2008.04.012

[28] Lima V, Burt B, Leibovitch I, Prabhakaran V, Goldberg RA, Selva D (2009) Orbital compartment syndrome: the ophthalmic surgical emergency. Surv Ophthalmol 54(4):441–449. 10.1016/j.survophthal.2009.04.005 19539832 10.1016/j.survophthal.2009.04.005

[29] Dos Santos JC, Gorla LFDO, Moreno R, Monnazzi MS, Pereira Filho VA, Gabrielli MFR, Gabrielli MAC (2024) Traumatic orbital compression syndromes: A comprehensive study into etiologies, intervention strategies, and clinical outcomes. J Craniofac Surg 35(5):1449–1455. 10.1097/SCS.0000000000010375 38838361 10.1097/SCS.0000000000010375

[30] Ujam A, Perry M (2016) Emergency management for orbital compartment syndrome-is decompression mandatory? Int J Oral Maxillofac Surg 45(11):1435–1437. 10.1016/j.ijom.2016.08.001 27575394 10.1016/j.ijom.2016.08.001

[31] Stewart CM, McDonald B, Clifford R, Norris JH (2016) Bilateral acute orbital compartment syndrome secondary to Richter syndrome: the ‘tulip’ sign. Clin Exp Ophthalmol 44(8):722–724. 10.1111/ceo.12759 27061968 10.1111/ceo.12759

[32] Reddy RP, Bodanapally UK, Shanmuganathan K, Byl G, Van der, Dreizin D, Katzman L, Shin RK (2015) Traumatic optic neuropathy: facial CT findings affecting visual acuity. Emerg Radiol 22(4):351–356. 10.1007/s10140-014-1292-3 25563705 10.1007/s10140-014-1292-3

